# The effect of moral distress experienced by intensive care nurses on end-of-life care attitudes and care behaviours: a single-centre cross-sectional and correlational study

**DOI:** 10.1186/s12912-026-04417-1

**Published:** 2026-02-12

**Authors:** Pakize Özyürek, İbrahim Kılıç, Ahmet Akarsu, Öznur Gürlek Kısacık

**Affiliations:** 1 Faculty of Health Sciences, Nursing Department, Afyonkarahisar Health Sciences University, Afyonkarahisar, 03200 Turkey; 2 Faculty of Veterinary Medicine, Biostatistics Department, Afyon Kocatepe University, Afyonkarahisar, 03200 Turkey; 3 Graduate Education Institute, Afyonkarahisar Health Sciences University, Afyonkarahisar, 03200 Turkey

**Keywords:** Moral distress, End-of-life care, Care behavior, Intensive care, Nurses, Attidute

## Abstract

**Objectives:**

This study aimed to evaluate the effect of moral distress experienced by intensive care nurses on their attitudes toward end-of-life care and their care behaviors.

**Methods:**

This cross-sectional and correlational study was conducted with 121 nurses working in intensive care units (ICUs). Data collection tools included the ICU Nurse Information Form, the Moral Distress Scale-Revised (MDS-R), the Caring Behaviors Inventory-24 (CBI-24), and the Nurses’Attitudes and Behaviors of Intensive Care Nurses Toward End-of-Life Care (EoLC) Scale.

**Results:**

The moral distress mean score of ICU nurses was 76.61 ± 22.72, attitudes and behaviors toward EoLC mean score 51.56 ± 10.39, and CBI-24 mean score was 122.72 ± 16.39.The correlation coefficients between the Distress subscale of the MDS-R and the mean scores of EoLC Attitudes were found to be positive and significant. (*r* = 0.200; *p* < 0.05; *p* = 0.028). The correlation coefficients between the CBI-24 and the EoLC Attitudes (*r* = 0.253, *p* < 0.01, *p* = 0.005) and Behaviors (*r* = 0.186, *p* < 0.05, *p* = 0.041) subscale were found to be positive and significant. Moral distress did not directly affect attitudes and behaviors toward EoLC, and care behaviour (*p* < 0.05).

**Conclusion:**

In this study, ICU nurses were found to experience moderate levels of moral distress, have good perceptions of caring behaviors, and exhibit moderate levels of attitudes and behaviors toward end-of-life care. However, moral distress was found to have no significant effect on their attitudes toward end-of-life care or on their caring behaviors.

**Clinical trial number:**

Not applicable.

## Introduction

Intensive care is a multidisciplinary and interprofessional specialty focused on the comprehensive management of patients with acute life-threatening organ dysfunction or those at risk of developing such conditions [1]. The intensive care unit (ICU) is characterized by advanced life-sustaining technologies, high clinical uncertainty, and complex decision-making processes. When ongoing treatment is perceived as non-beneficial, difficult decisions regarding the continuation, limitation, or withdrawal of life-sustaining interventions become unavoidable [2, 3]. Such decisions place intensive care teams in ethically challenging situations, particularly in the context of end-of-life care.

Moral distress is a psychological condition that arises when healthcare professionals encounter situations that conflict with their ethical values [4, 5]. In the ICU setting, moral distress frequently emerges during end-of-life care when clinicians are unable to act in accordance with what they believe to be ethically appropriate due to personal, professional, or organizational constraints. Moral distress may lead to significant emotional consequences, including frustration, guilt, burnout, and emotional exhaustion, thereby impairing decision-making capacity and compromising the quality of care [6]. As an occupational stressor, moral distress can affect multiple dimensions of professional competence, particularly in complex medical environments such as intensive care units [7, 8]. Understanding and addressing moral distress is therefore vital not only for safeguarding nurses’ psychological well-being but also for maintaining standards of end-of-life care and ensuring organizational stability [6].

Within the multidisciplinary team, intensive care nurses tend to experience moral distress more intensely because they closely witness patients’ struggles between life and death and are frequently involved in end-of-life decision-making processes [7, 9]. The literature suggests that moral distress arises from the interaction between internal and external conditions, with internal conditions related to psychological responses and external conditions associated with the work environment. In contemporary healthcare systems, moral distress has emerged as a significant issue that can be experienced by healthcare team members and may threaten the integrity of the care provided [10].

Mortality rates are high in ICUs, and ICU nurses deal with death on a daily basis. Although preparing for the dying process is challenging, ICU nurses’ responses and attitudes toward death may vary depending on their previous experiences [7]. For ICU nurses, working with insufficient numbers of nurses and limited access to resources are among the significant causes of moral distress. However, the most frequently emphasized and debated source of moral distress in the nursing literature is the ethical and clinical dilemmas related to end-of-life care [11]. Additionally, ICU nurses and other healthcare professionals tend to perceive end-of-life care as futile treatment [12]. This situation is further highlighted by the fact that nurses’ efforts to act in accordance with ethical values in their care decisions are hampered by patient conditions and environmental constraints [11, 13]. Under these conditions, the concept of moral distress has become increasingly prevalent and salient, emerging as a significant concern that threatens both nurses’ psychological well-being and the quality of care [1]. Moral distress in nurses is known to lead to anger, frustration, and emotional suffering, contribute to disengagement from the profession, and negatively affect the quality of patient care [10].

ICU nurses experience negative emotions such as sadness, grief, helplessness, fear, and hopelessness when caring for patients who are approaching death. Consequently, they report not wanting to provide care, experiencing difficulty discussing the concept of death with patients and their families, and feeling inadequate in this regard [14]. ICU nurses’ attitudes toward death affect the support provided to family members and the quality of end-of-life care [7].Consequently, ICU nurses’ attitudes toward end-of-life care may be related to the core competencies of their care behaviors.

If moral distress is not identified in a timely manner and managed appropriately, it can lead to serious problems in patient care processes. This situation may affect not only the quality of care but also patient safety [15]. As nurses encounter an increasing number of situations involving moral distress, there is a possibility that they may experience adverse physical, psychological, emotional and spiritual changes associated with compassion fatigue and occupational burnout [5]. In intensive care settings, where ethically challenging situations are frequent, moral distress may influence nurses’ attitudes toward end-of-life care as well as their perceived care behaviours [16]. Previous studies have primarily examined either moral distress or end-of-life care attitudes among intensive care nurses as separate phenomena. Previous studies conducted in Turkey have examined either moral distress or end-of-life care and attitudes of ICU nurses separately. A review of these studies indicates that moral distress levels were low [10, 17, 18], whereas attitudes toward end-of-life care were found to be at a moderate level [19, 20].

However, there is a limited body of research simultaneously examining these relationships among intensive care nurses, underscoring the need for a framework-based investigation. While some studies have identified an association between moral distress and care behaviors [16], others have reported no such relationship [21, 22]. This study aims to examine the impact of moral distress experienced by nurses working in intensive care settings on their attitudes toward end-of-life care and their care behaviors. By addressing these interrelated constructs simultaneously, the study seeks to contribute to a more comprehensive understanding of how moral distress influences end-of-life care practices in intensive care settings and to inform the development of strategies aimed at managing moral distress and improving the quality of care.

On the basis of these informationsin the literature, the following research questions were developed:

1. What is the level of moral distress experienced by ICU nurses?
2. What are ICU nurses’ attitudes toward end-of-life care?
3. How do ICU nurses perceive their care behaviors?
4. Is moral distress associated with ICU nurses’ attitudes toward end-of-life care and care behaviors?

In addition, the following hypotheses were tested:

### H_1_

There is a significant relationship between the moral distress experienced by intensive care nurses and their attitudes toward end-of-life care.

### H_2_

There is a significant relationship between the moral distress experienced by intensive care nurses and their caring behaviors.

## Method

### Study design

This study aimed to examine the relationships between data obtained from three validated questionnaires within a single population at one point in time. The nature of the study questions required a non-experimental, descriptive, and quantitative research design with a cross-sectional and correlational approach.

### Sample and participant

The study data were collected between January 2023 and April 2023 in four surgical and five medical ICUs at the health application and research center. The total number of beds in ICUs is 103. The population of this study consisted of 129 Turkish-speaking nurses working in four surgical and five medical ICUs of a university hospital in Turkey. In accordance with the results of analogous studies cited in the existing literature, no sampling was conducted as the objective of the study was to reach the entire population [10, 18]. The final sample included 121 ICU nurses who had been working in the ICUs and were willing to participate in the study. Regarding employment status, all ICU nurses were full-time employed nurses; therefore, employment status did not vary within the sample and was not examined as a separate variable.

### Inclusion and exclusion criteria

The inclusion criteria stipulated that in order to participate in the study, ICU nursing staff were required to have a minimum of six months of professional experience in ICUs, in addition to being willing to participate voluntarily.

The exclusion criteria encompassed ICU nurses who met one or more of the following criteria: those who were not enrolled in the ICUs, those with less than six months of professional experience, and those who declined to participate. Eight ICU nurses were excluded from the study due to incomplete questionnaire responses, unwillingness to participate, or being on leave due to illness.

### Data collection procedure

The researchers informed ICU nurses about the purpose of the study through face-to-face communication and obtained their voluntary consent. Data collection was conducted during break times outside of nursing care hours. To ensure confidentiality, the data collection forms were completed anonymously. After completion, the questionnaires were returned to the researchers in sealed envelopes during the next visit. All collected data were securely stored in accordance with confidentiality and data protection principles. The average time required to complete the questionnaire was 10–15 min, and the participation rate among ICU nurses was 93.7%.

### Data collection instruments

The data collection instruments used in this study included the ICU Nurses Information Form, the the Moral Distress Scale-Revised (MDS-R), the Caring Behaviors Inventory-24, and the Attitudes and Behaviors of Intensive Care Nurses Toward End-of-Life Care Scale.

### Nurse information form

The form consists of nine questions designed to determine the individual (age, gender, education, etc.) and professional characteristics (unit, years of experience, number of patients cared for, etc.) of ICU nurses.

### Moral distress scale-revised (MDS-R)

The MDS-R, developed by Corley et al. (2001), is designed to measure moral distress in ICU nurses [23]. The Turkish adaptation was conducted by Karagözoğlu et al. (2017) [18], . The scale consists of 21 items rated on a five-point Likert scale. For the frequency dimension, responses range from “never” (0) to “very often” (4), and for the distress dimension, responses range from “none” (0) to “very much” (4), comprising two subscales [18]. The total score ranges from 0 to 336, with higher scores indicating greater levels of moral distress. The original scale demonstrated a Cronbach’s alpha of 0.88 and a test–retest correlation of 0.58. For the Turkish version, Cronbach’s alpha was 0.85, and the test–retest correlations were 0.85 and 0.82, respectively [18]. In this study, the Cronbach’s alpha for the MDS-R was 0.88.

### Caring behaviors inventory-24 (CBI-24)

The scale was designed to assess the nursing care process, allowing nurses to self-evaluate and compare their perceptions with those of patients [24, 25]. The Turkish validity and reliability tests were conducted by Kurşun and Kanan (2012) [26]. The CBI-24 consists of 24 items across four subscales: assurance, knowledge–skill, respectfulness, and connectedness. Responses are rated on a six-point Likert scale (never = 1; always = 6) [26]. Total scores range from 24 to 144, with higher subscale and total scores indicating higher perceived quality of care by nurses or patients. The internal consistency of the Turkish version ranged from 0.82 to 0.92 for subscales and 0.96 for the total scale [26]. In this study, the Cronbach’s alpha for the Caring Behaviors Inventory for nurses was 0.89.

### The nurses’attitudes and behaviors of intensive care nurses toward end-of-life care (EoLC) scale

This scale, developed by Zomorodi and Lynn (2010), evaluates ICU nurses’ attitudes and behaviors regarding the end-of-life care process [27]. The scale consists of 16 items across two subscales and uses a five-point Likert scoring system. The original scale demonstrated a Cronbach’s alpha of 0.78. The Turkish adaptation by Yalçınkaya (2016) reported a Cronbach’s alpha of 0.70 [20, 28]. In this study, the Cronbach’s alpha for the scale as 0.82.

### Data analysis

The study data were analysed using SPSS version 22.0 (Armonk, NY: IBM Corp.). The Kolmogorov–Smirnov test was used to assess the normality of the data distribution. Descriptive statistics, such as frequency, percentage, mean and standard deviation, were employed to summarise the data. Independent sample t-tests and analysis of variance (ANOVA) were employed to determine relationships and statistical differences between variables. Pearson correlation and multiple linear regression analyses were used to evaluate the relationships between total and subscale scores of the instruments. Correlation coefficients are generally interpreted as weak when ranging from 0.10 to 0.29, moderate when ranging from 0.30 to 0.49, and strong when 0.50 or higher [29]. A significance level of *p* < 0.05 was used for all statistical analyses.

### Ethical considerations

Written approval for the study was obtained from the Afyonkarahisar Health Sciences University Non-interventional Scientific Research Ethics Committee (Date: 02/12/2022; Number: 2022/579) and the hospital administrators where the study was conducted (Date: 04/11/2022; Number: E.119927). Permission to use the Turkish versions of the scales was obtained from the authors via email. Informed consent was obtained from all participants prior to their inclusion in the study. In addition, the ICU nurses were clearly informed about the purpose, scope, and were assured that their participation was voluntary and that they could withdraw at any time without any consequences. All procedures were conducted in accordance with the relevant ethics committee approval and the principles of the Declaration of Helsinki.

## Results

### Participants’ information

Of the ICU nurses, 66.9% were female and 33.1% were male, with a mean age of 28.60 ± 4.54 years. 66.9% of ICU nurses had a bachelor’s degree and 33.1% had a vocational high school or associate’s degree programs. Among the ICU nurses, 56.2% worked in medical ICUs and 43.8% in surgical ICUs, with a mean total working experience of 6.65 ± 4.48 years.

The mean duration of ICU experience was 5.00 ± 3.44 years. The average number of patients cared for per shift was 4.16 ± 1.62 (Table 1).

**Table 1:** Nurses’ ındividual and professional characteristics (*n* = 121)

Variables	Group	*n*	%
Age (year)	≤ 26 27–30 ≥ 31	41 53 27	33.9 43.8 22.3
Sex	Female Male	81 40	66.9 33.1
Education Level	Vocational/associate degree Bachelor’s degree	40 81	33.1 66.9
Marital status	Married Single	67 54	55.4 44.6
ICU type	Surgical Medical	53 68	43.8 56.2
Total Years of Nursing Experience	≤ 5 6–10 ≥ 11	67 40 14	55.4 33.1 11.6
Average Patients per Nurse	≤ 3 4–5 ≥ 6	76 17 28	62.8 14.0 23.1
**Total**		121	100

ICU: Intensive Care Unit

### MDS-R scores of ICU nurses

The mean score of ICU nurses on the “frequency” subscale of the MDS-R was 31.45 ± 14.36, while the mean score on the “distress” subscale was 45.09 ± 15.47. The overall mean score was 76.61 ± 22.72 (out of a possible range of 0–336) (Table 2).

**Table 2:** Descriptive analysis results of the scales and their subscales

Scale	Subscales	Min-maks.	Mean	SD
Moral Distress Scale-Revised	Frequency	6–73	31.45	14.36
	Distress	9–80	45.09	15.47
	**Total**	30–126	76.61	22.72
Caring Behaviors Inventory-24	Assurance	9–48	42.65	5.54
	Knowledge–Skill	6–30	27.47	3.58
	Respectfulness	6–36	31.61	4.83
	Connectedness	7–30	25.99	3.83
	**Total**	28–144	122.72	16.39
The Nurses’Attitudes and Behaviors of Intensive Care Nurses Toward End-of-Life Care	Attitudes	10–50	33.96	6.69
	Behaviors	6–30	17.5	5.52
	**Total**	20–80	51.56	10.39

SD: Standard Deviation

### CBI-24 scores of ICU nurses

The mean score of ICU nurses on the “Assurance” subscale of CBI-24 was 42.65 ± 5.54, on the “Knowledge-Skill” subscale was 27.47 ± 3.58, on the “Respectful” subscale was 31.61 ± 4.83, and on the “Connectedness” subscale was 25.99 ± 3.83. The overall mean score of the scale was 122.72 ± 16.39 (Table 2).

### Nurses’ attitudes and behaviours towards the EoLC scale scores

The mean score for the “Attitude” subscale of Nurses’ Attitudes and Behaviours towards the EoLC Scale was 33.96 ± 6.69, the mean score for the “Behavior” subscale was 17.5 ± 5.52, and the overall mean score was 51.56 ± 10.39 (Table 2).

### The relationship between the individual and professional characteristicso of ICU nurses and the total and subscale scores of the scales

The correlation between ICU nurses’ individual and professional characteristics—such as age, gender, marital status, education level, type of ICU worked in, years of experience, and the number of patients cared for per shift—and the mean scale scores was examined. A statistically significant relationship was found between the type of ICU and the mean CBI-24 total score, as well as between the number of patients cared for per shift and the mean score of the attitude subscale of the Nurses’ Attitudes and Behaviours towards the EoLC Scale (*p* < 0.05) (Table 3).

**Table 3:** Comparison of nurses’ individual and professional characteristics with the mean scores of the scales and subscales

Variables	*n*	CBI-24	MDS-*R*	Attitudes and Behaviors of Toward EoLC
		Mean ± SD	Mean ± SD	Mean ± SD
**Age (year)**				
≤ 26 27–30 ≥ 31 F test/p	41 53 27	122.80 ± 21.29 130.15 ± 13.25 130.44 ± 11.58 2.884/0.060	74.46 ± 15.99 76.30 ± 25.23 80.48 ± 26.34 0.575/0.564	50.26 ± 11.28 51.98 ± 10.62 52.70 ± 8.53 0.519/0.596
**Sex**				
Female Male t-testi/p	81 40	5.38 ± 0.56 5.24 ± 0.80 1.14/0.25	1.85 ± 0.51 1.77 ± 0.60 0.75/0.47	3.17 ± 0.63 3.15 ± 0.76 0.15/0.88
**Education Level**				
Health vocational high school Bachelor’s degree t-testi/p	40 81	5.30 ± 0.60 5.33 ± 0.72 -0.20/0.84	1.81 ± 0.47 1.83 ± 0.57 -0.19/0.84	3.08 ± 0.69 3.20 ± 0.66 − 0.96/0.33
**Marital Status**				
Married Single t-testi/p	67 54	5.38 ± 0.56 5.24 ± 0.80 1.14/0.25	1.85 ± 0.58 1.78 ± 0.49 0.71/0.47	3.15 ± 0.62 3.17 ± 0.74 -0.16/0.86
**ICU type**				
Surgical Medical t-testi/p	53 68	5.48 ± 0.42 5.19 ± 0.81 2.56/0.01	1.86 ± 0.59 1.78 ± 0.49 0.81/0.42	3.25 ± 0.66 3.09 ± 0.68 1.23/0.21
**Years of Nursing Experience**				
≤5 6–10 ≥ 11 F test/p	67 40 14	126.02 ± 18.47 129.42 ± 14.43 131.00 ± 9.51 0.850/0.430	74.76 ± 22.55 80.62 ± 22.54 74.00 ± 24.20 0.937/0.395	51.44 ± 11.30 50.52 ± 8.44 55.07 ± 10.85 1.00/0.371
**Average Patients per Nurse**				
≤3 4–5 ≥ 6 F test/p	76 17 28	126.78 ± 17.52 129.70 ± 13.86 129.07 ± 14.88 0.338/0.714	77.19 ± 22.06 82.11 ± 22.20 71.67 ± 24.60 1.187/0.309	50.88 ± 10.31 51.11 ± 11.05 50.03 ± 9.43 2.988/0.054

SD: Standard Deviation; MDS-R: Moral Distress Scale-Revised; CBI-24: Caring Behaviors Inventory-24; EoLC: End-of-Life Care

### The relationship between the total and subscale scores of the scales

The distress subscale of the MDS-R showed a weak but statistically significant positive correlation with the attitude subscale of the Nurses’ Attitudes and Behaviours toward the EoLC Scale (*r* = 0.200, *p* = 0.028). In addition, CBI-24 scores were weakly and positively correlated with both the attitude subscale (*r* = 0.253, *p* = 0.005) and the behaviors subscale (*r* = 0.186, *p* = 0.041) of the EoLC Scale.No significant differences were found among the correlation coefficients of the other subscales (*p* > 0.05) (Table 4).

**Table 4:** Correlation coefficients among the scales

Scales	CBI-24	MDS-*R* Frequency	MDS-*R* Distress	Attitudes Toward EoLC	Behaviors of Toward EoLC
CBI-24	1	-0.0740	0.0830	0.253**	0.0186*
MDS-R; Frequency	-0.740	1	0.0167	-0.60	0.0370
MDS-R; Distress	0.830	0.0167	1	0.200*	0.0110
Total MDS-R	0.009	1	1	0.091	0.096
Attitudes of Toward EoLC	0.253	-0.600	0.200*	1	0.442**
Behaviors of Toward EoLC	0.186	0.037	0.110	0.442**	1

*P* < 0.05* *P* < 0.01**; MDS-R: Moral Distress Scale-Revised; CBI-24:Caring Behaviors Inventory-24; EoLC: End-of-Life Care

### The effect of MDS-R on CBI-24, and attitudes and behaviours towards the EoLC scale

When Table 5 is examined, none of the coefficients were found to be statistically significant, and the multiple linear regression model can be expressed as:

**Table 5:** The effect of moral distress on attidute and behaviors of toward EoLC and care behaviors

Variables	Coefficient	Standard Error	SE(B)	t	*p*
Constant	65.721	17.588		3.737	0.000
**Attitudes and Behaviors of Toward EoLC**	0.267	0.08	0.121	1.281	0.203
CBI-24	-0.023	0.132	-0.016	-0.174	0.862

*R* = 118; R^2^= 0.014; F = 0.835; *p* = 0.437; CBI-24:Caring Behaviors Inventory-24; SE(B): Standard Error of B

Moral distress = 65.721 + 0.2367*1 + 0.023*2.

According to the multiple linear regression model, an increase in attitudes and behaviours towards the EoLC raised the level of moral distress by 0.267 units; however, this increase was not statistically significant. Although higher levels of caring behaviors were found to decrease moral distress, this decrease was also not statistically significant (*p* < 0.05).

## Discussion

The results of this study showed that there was no statistically significant relationship between moral distress experienced by ICU nurses and their attitudes toward end-of-life care or their caring behaviors. However, ICU nurses reported moderate levels of moral distress and attitudes toward end-of-life care, while their perceptions of caring behaviors were at a good level. These results suggest that, in intensive care settings, nurses may be able to maintain their caring behaviors despite experiencing moderate moral distress.

### MD levels and predictors of ICU nurses

In this study, the overall average MDS-R total score of ICU nurses was moderate. This was consistent with studies investigating the moral distress levels of ICU nurses [8, 30, 31]. In some studies conducted in Turkey, nurses’ moral distress scores were low [10, 17, 18].

This difference may be attributed to personal characteristics, the use of different measurement tools, or variations in institutional and working conditions [8]. However, a common aspect of the aforementioned studies is that the distress experienced by nurses in relation to moral distress is generally associated with end-of-life care for terminal patients and interventions perceived as having no benefit. The most common sources of moral distress in ICUs include futile and aggressive treatments that prolong the dying process in terminal patients, inability to control pain symptoms, practices that disregard patient autonomy, failure to act honestly with patients and their relatives, and insufficiently informed consent [32].

This study showed that gender, age, professional experience, and educational level did not have a significant effect on the moral distress experienced by ICU nurses. This results suggested that moral distress was influenced more by situational and contextual factors than by demographic characteristics. In contrast, a significant relationship was found between the number of patients cared for during a single shift and moral distress scores, indicating that workload and care intensity may have been important determinants of moral distress. These results were consistent with those of previous studies [10, 33].

There are also studies in the literature emphasizing a relationship between moral distress and ICU nurses’ professional experience. Some studies report that ICU nurses with more professional experience exhibit higher levels of moral distress [31, 32] while others indicate lower levels of moral distress among more experienced ICU nurses [30, 34, 35].

In our study, younger ICU nurses with less professional experience had higher total moral distress scores, although this was not statistically significant. This may be related to the fact that younger ICU nurses, who are expected to take on significant responsibilities in critical care settings, are more likely to recognize ethical dilemmas and challenges, make appropriate decisions, and manage problems despite having limited knowledge and skills. Conversely, increased experience and enhanced problem-solving abilities with older nurses may lead to greater sensitivity, which can result in lower levels of moral distress among nurses [32].

### The ICU nurses’ perceptions of caring behaviors

This study found that ICU nurses’ overall perception of care practices was observed to be very positive (85.2%). These results were consistent with previous studies conducted among ICU nurses, such as those by Ahmed et al. and Hwang et al. [16, 27, 28, 36], while they were observed to have a more positive perception than those reported in studies by Lukmanulhakim et al. and Taylan et al. [37, 38].

In this study, ICU nurses’ perceptions of caring behaviors were not shaped by socio-demographic (e.g., gender, educational level, marital status) and professional characteristics (e.g., years in the profession, working hours). Contrary to these results, some studies reported that socio-demographic and professional characteristics affect caring behavior [16, 39, 40]. The observed discrepancies between the present results and those of previous studies may be attributable to differences in healthcare settings, cultural contexts, or methodological approaches.

### The ICU nurses’attitudes and behaviors of EoLC

In this study, ICU nurses’ attitudes and behaviors toward EoLC were found to be at a moderate level, similar to those reported in previous studies [19, 20]. A possible reason why ICU nurses in the present study did not demonstrate the desired level of attitudes and behaviors toward EoLC may be their frequent exposure to end-of-life cases compared to nurses in other clinical settings. This explanation was consistent with the study by Teixeira Cardoso et al. (2021), where nurses who were more exposed to death had negative attitudes toward end-of-life care [41].

In the present study, ICU nurses reported that asking questions to identify patients’ care needs made them feel more comfortable. In line with their responsibilities and nursing roles, nurses demonstrated a positive attitude in communicating their observations and knowledge to other healthcare professionals and collaborating with the family and their team to determine and verify the best treatment and care.

It was determined that nurses were unable to demonstrate collaborative attitudes and behaviors with patients and their families regarding the spiritual and informational aspects of the dying process. Hamdan et al. (2023) showed that ICU nurses had insufficient knowledge of palliative care and inappropriate attitudes [42]. The weakest attitudes of nurses were observed in communication with dying patients, particularly in discussing death [42].

ICU nurses should play an active role in ensuring that patients receiving end-of-life care are provided with comfort-focused rather than treatment-focused care. ICU nurses should help the families of patients approaching the end of life understand what they are feeling and support them in actively preparing for the dying process [43]. Therefore, nursing education institutions need to focus on end-of-life care in nursing curricula [42]. Providing end-of-life care that encompasses not only technical and physical nursing care but also spiritual care involving compassion and mercy is considered to hold important value for professional nursing and may have a decisive impact on the quality of care delivered in intensive care units.

No significant relationships were found between ICU nurses’ demographic and professional characteristics and their overall attitudes and behaviors toward EoLC, except for a significant association between patient load per shift and the score on the ‘attitude’ subscale. These results suggest that situational factors, such as workload, may influence ICU nurses’ attitudes and behaviors toward EoLC more than fixed demographic or professional characteristics, which is consistent with some previous studies [44, 45], while other studies have reported different results [13, 19, 20].The differences in results may be due to the use of different scales toward EoLC. Previous studies investigating behaviors and attitudes toward EoLC indicate that the understanding of care is not consistent among nurses in different societies. Cultural differences may influence how care behaviors are expressed [46].

### The Correlation between moral distress and attitudes and behaviors toward EoLC and perceptions of caring behaviors

In this study, no statistically significant relationship was observed between the mean moral distress scores of ICU nurses and their mean scores on attitudes and behaviors toward EoLC or CBI-24. In other words, experiencing moral distress did not appear to affect nurses’ attitudes toward EoLC or their perceptions of care behaviors. It was observed that as ICU nurses’ care behaviors became more positive, their levels of moral distress tended to decrease; however, this decrease was not statistically significant. This indicates that moral distress did not have a negative impact on nurses’ perceptions of care behaviors; on the contrary, it suggested that they maintain professional attitudes and fulfill their care responsibilities. Safari et al. (2024) found that no statistically significant relationship was found between moral distress and clinical care quality [15]. In the study by Mahmoodzadeh et al. (2018), a weak negative correlation was observed between moral distress and care behaviors [46].

Although moral distress did not show a direct association with attitudes toward end-of-life care, a weak positive association was identified between the distress subscale of the MDS-R and attitudes toward EoLC. In the study by Rezaei et al. (2023), no direct relationship was observed between attitudes toward EoLC and moral distress [47]. Attitudes toward end-of-life care are influenced by individual factors, such as cultural norms, educational level, and personal values. This may explain why moral distress did not significantly affect these attitudes [7]. The impact of moral distress is also shaped by factors such as nurses’ ethical awareness and organizational support; in settings where these factors are strong, the negative effects of moral distress may be mitigated [47]. It can be suggested that as the level of moral distress decreases, care behaviors and attitudes may improve; however, further research is necessary to explore and address this relationship. It could be suggested that as the level of moral distress decreases, care behaviors and attitudes might improve. However, further research is necessary to explore and address this relationship [47]. Nurse managers should pay adequate attention to reducing moral distress and thereby improving care quality. In this context, appropriate leadership practices can be implemented, such as providing training to nurses by organizing ethics workshops, providing counseling, changing work units, reviewing procedures and guidelines, reducing working hours, and informing nurses about moral distress and its consequences [8].

### Limitation of study

This study included only nurses working in adult ICUs at a hospital in one province in Turkey. Therefore, the results cannot be generalized to all ICU nurses. Additionally, the use of a survey-based design and the reliance on data collected solely from nurses’ self-reported responses constitute another limitation of this study. Consequently, it was not possible to explore in depth the impact of moral distress on attitudes toward EoLC and care behaviors using qualitative methods.

### Recommendations

Future qualitative or mixed-methods studies are needed to explore more deeply how ICU nurses’ lived experiences and narratives may influence their attitudes toward end-of-life care and their caregiving behaviors in the context of moral distress. Furthermore, considering the relationship between moral distress and care behaviors, nurse managers can strengthen care practices by developing strategies aimed at helping nurses, particularly those working in critical units, cope with and reduce moral distress.

## Data Availability

The datasets generated and analysed during the current study are available from the corresponding author on reasonable request.
